# 

**Published:** 1897-03

**Authors:** 


### CONTENTS—FEBRUARY, 1897.—VOL. XII., NO. 9.

ORIGINAL CONTRIBUTIONS—
Consumption in San Antonio. R. Menger, M.D. . . . . . . . . . . . . . . 475
Chronic Diffuse Nephritis without Exudation. Joe S. Wooten, B.Sc., M.D., 479
CORRESPONDENCE—
For the Benefit of the Unfortunate Young Man. F. B. McRae, M.D.; J. W. Cole, M.D.; J. S. W., M.D.; Benj. Frenkel, M.D.; W. H. Lancaster, M.D. . . . . . . . . . . . . . . . . . . . . . . . . . . . . 488–496
Spider Bite. A. H. Schenk, M.D. . . . . . . . . . . . . . . . . . . . 493
The Texas Health College Evil Again . . . . . . . . . . . . . . . . . . 496
SOCIETY NOTES—
Texas State Medical Association . . . . . . . . . . . . . . . . . . . . 498
ABSTRACTS AND SELECTIONS—
Puerperal Eclampsia—Its Etiology and Treatment . . . . . . . . . . . 500
Vaginal Ligation of the Uterine Arterias for Uterine Fibromata . . . . . . 502
Notes on Some of the Newer Remedies Used in Diseases of the Skin . . . . 503
A Department of Public Health in the U. S. Government . . . . . . . . . 505
The Doctor's Wife . . . . . . . . . . . . . . . . . . . . . . . . . . 506
EDITORIAL—
Suicide . . . . . . . . . . . . . . . . . . . . . . . . . . . . . . . 507
Penny Wisdom—Mistaken Philanthropy . . . . . . . . . . . . . . . . . 511
A State Hospital . . . . . . . . . . . . . . . . . . . . . . . . . . 512
The Meharry Medical College . . . . . . . . . . . . . . . . . . . . . 514
The Association's Bill . . . . . . . . . . . . . . . . . . . . . . . . 514
MEDICAL NEWS AND MISCELLANY . . . . . . . . . . . . . . . . . . . 517–522
BOOK NOTICES . . . . . . . . . . . . . . . . . . . . . . . . . . . 522–524
PUBLISHERS' NOTES . . . . . . . . . . . . . . . . . . . . . . . . 524–532

